# The Morphological Features and Biology of a Relict and Endangered Woody Plant Species: *Chamaedaphne calyculata* (L.) Moench (Ericaceae)

**DOI:** 10.3390/plants8050129

**Published:** 2019-05-15

**Authors:** Anna Źróbek-Sokolnik, Piotr Dynowski, Mieczysława Aldona Fenyk

**Affiliations:** 1 Department of Botany and Nature Protection, University of Warmia and Mazury in Olsztyn, Plac Łódzki 1, 10-727 Olsztyn, Poland; aldi@uwm.edu.pl; 2 Institute of Geography and Land Management, University of Warmia and Mazury in Olsztyn, Prawocheńskiego Str. 15, 10-720 Olsztyn, Poland; piotr@jezioro.com.pl

**Keywords:** Ericaceae, leaf morphometrics, protected plant, seed germination

## Abstract

*Chamaedaphne calyculata* (L.) Moench is a rare post-glacial relic, which reaches the south-western boundary of its European range in Poland. However, little is known about the morphology and biology of this species. In this study a biometric analysis of assimilating leaves and general morphological appearance was carried out; flowering, fruiting, and seed production in a natural site were described and the ability of seed germination was determined after varied seed storage time at 0–2, 2–4, 6–8, and 21–23 °C. A quite large intrapopulation variability was found as regards the features under analysis. The most varied features included the length of the petiole, followed by the leaf blade area, while the length to the width of the leaf blade was the least varied feature. *C. calyculata* flowered abundantly and about 50% developed flowers turned into fruits. On average there were 56 seeds per fruit with a predominance of mature seeds. The length of the seed storage time had a significant effect on all parameters of the germination process. The temperature at which the seeds were stored, apart from the time to maximum germination parameter, had a significant effect on other parameters of the germination process. Interactions between the seeds storage time and temperature factors were observed only for germination percentage, germination index, and germination index rate parameters. The ability of seeds to germinate, non-creation of the seed bank and other results of the research provide new information necessary for planning and carrying out conservation procedures (including active conservation in ex situ conditions).

## 1. Introduction

In Poland, rare and endangered plants account for about 15% of all vascular flora species (considering native species and archeophytes) [1]. The largest group comprises taxa occurring in boreal and arctic zones.

*Chamaedaphne calyculata* (L.) Moench (leatherleaf) is a wintergreen dwarf shrub of the Ericaceae family, growing in the boreal and subarctic zone of Europe, Asia, and North America. It can be found in highly moisturized communities of marshy coniferous forests and high moors of acid soils (pH below 5). *C. calyculata* is considered a typical species for the set of *Oxycocco-Empetrion hermaphroditi* communities (Nordh. 1936) R.Tx. 1937, covering high moors in subarctic-boreal zone of Europe. In Poland, it is a rare post-glacial relic (currently growing at 10 sites and placed under full legal protection), which reaches here the south-western boundary of its European range and creates highly isolated Central European populations [2,3,4]. The majority of *C. calyculata* sites in Poland are located in the northern parts of the country, in the lake district belt and in the Mazovian-Podlasie Lowland. This species can be found mainly in non-forest high moors, in the *Sphangnetum magellanicii* community (Malc. 1929) (Kästner et Flössner 1933) in the marshy coniferous forest, *Vaccinio uliginosi-Pinetum sylvestris* (Kleist 1929) [2,5,6].

Its flowers are white, single, up to 4 mm long, hanging in the leaf axils on short stalks. They form a one-sided, panicle-type inflorescence. The flowering period is between April and June. The fruit takes the form of a spherical, distended capsule, bursting with five valves [7]. Leatherleaf seeds are small (up to about 1 mm length and about 0.12 mg), smooth and light-brown. They are dispersed when the dry capsules open at the end of the growing season. The seeds have a corky wing, which enables them to float on water and also facilitates wind dispersal [8].

Populations inhabiting individual sites in Central Europe differ in the number of individuals, the nature of their growth, density, abundance of flowering, and the intensity of fruiting. In open areas, leatherleaf often forms thick clusters and in the ecotone zone between the forest and the moors—streak-shaped scrubs, while in marshy coniferous forests it occurs most frequently as single shoots [2]. Kruszelnicki [2] reports that in well-lit places, *C. calyculata* abundantly flowers and bears fruit. Wróblewska [3], however, observed that in two of the marginal populations in Poland (Jesionowe Góry Resrve and Kampinos National park) flowering and fruit set were sporadic or absent over several years. Other observations in these populations shown abundant flowering but low fruit set. Furthermore, the percentage of developed seeds, as well as germinability and seed variability were low [9]. For that reason, it is believed that leatherleaf in Central Europe is mainly propagated vegetatively through rooting shoots and producing root suckers, while the share of generative propagation is much lower [2]. However, the results of Wróblewska’s work [3,4] showed that *C. calyculata* specimens growing in Poland were characterized by high genetic variation. This suggests the possibility of significant efficiency of generative reproduction in the survival strategy of this species. Against the background of a very rich literature on germination and breaking dormancy of seeds, data on the biology of this species, especially the reproduction and germination of leatherleaf seeds, are particularly poor. Densmore [10] examined, among others, the effect of day length, stratification and temperatures on breaking dormancy and germination of seeds of *C. calyculata* growing in Alaska. Selected aspects of leatherleaf reproduction, among others the level of individual fertility and germination of seeds from Polish populations growing in the Drawa National Park, were described by Malinowska et al. [9].

Currently, *C. calyculata* is endangered because of industrial development, environmental pollution and climatic change. The direct and greatest threat to the described taxon is the destruction of its natural biocenosis. The main reason is the drainage of peat bogs and their surroundings, which is the most often the consequence of human activity, such as intensive land reclamation and peat mining [2]. Climate conditions also have a limiting effect on the development of populations of this plant. Hot, dry summers cause a decrease of the level of ground waters, and cold, snowless winters cause the winterkill of seedlings [11,12,13]. The succession changes which lead to the encroaching of trees and expansion of deciduous species causing shadowing also adversely affect *C. calyculata* populations. Sphagnum fires are also a threat for habitat of leatherleaf [2].

Active in situ plant conservation on is connected with undertaking protective tasks consisting in attempts to maintain habitats [2]. Tasks related to active ex situ conservation include replacements of specimens from highly threatened populations to nearby well-preserved peat bogs [2], breeding in botanical gardens and arboretums [14] and successful attempts to multiply this species from vegetative parts in in vitro cultures [15,16]. Due to the lack of deeper information on the sprouting of leatherleaf seeds, *C. calyculata* seeds and seedlings have not been used for in vitro reproduction until now, although the literature data present valence of this type of plant material in propagation of endangered Ericaceae species (e.g., [17,18,19]). The development of methods for obtaining seedlings and embryos would allow them to be used in in vitro cultures for reproduction of the mother plant and then reintroduction. It should also be added that it is also a medical plant-a poultice of the leaves of *C. calyculata* has been applied to inflammations and an infusion of the leaves has been used to treat fevers in traditional medicine of native American [20]. Furthermore, extracts of its leaves and flowers contain substances of a flavonoid nature [21,22,23]. Leatherleaf has also decorative value as an ornamental shrub, which is sold by gardening nurseries in Poland, Europe and other countries around the world. Summarizing, explanation of issues related to *C. calyculata* generative breeding is a potential source of knowledge for plant biologists and biotechnologists. Knowledge about sprouting of leatherleaf seeds can also be used in forestry, nursery, gardening and landscaping architecture.

The aim of this study was: (1) description the condition of *C. calyculata* at a new site based on biometric analysis of assimilating leaves and general morphological appearance, (2) determination the characteristic of flowering, fruiting and seed production of *C. calyculata* in a natural site including initial characterization of seed heteromorphism (confirmation or not confirmation of literature data), (3) determination the possibility of generative propagation this species under laboratory conditions and explanation of the following issues: the level of individual fertility of *C. calyculata*, the effect of storage conditions on germination, the possibility of creating a seed bank, in the context of planning of the tasks associated with active plant species conservation.

## 2. Materials and Methods

### 2.1. Study Area

**Figure 1:**
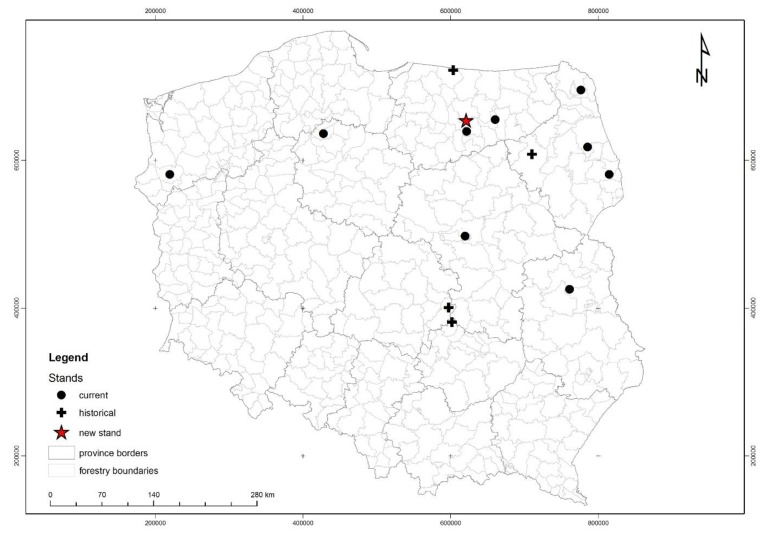
Distribution of the sites of *Chamaedaphne calyculata* in Poland [2,24,25] with the new site designated with star.

**Figure 2:**
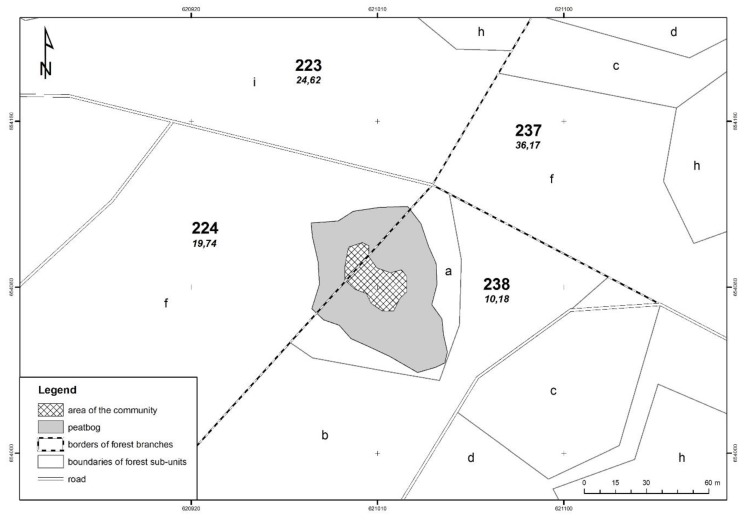
The range of plant community with *Chamaedaphne calyculata* at the new site near Nerwik village.

**Figure 3:**
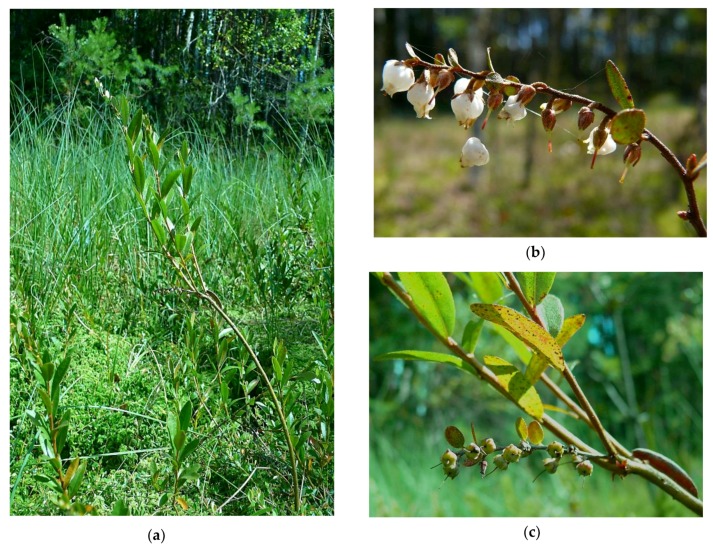
*Chamaedaphne calyculata* at the new site near Nerwik village. (**a**) General growth form, leaves and branches. (**b**) Flowers. (**c**) Unripe fruits.

**Figure 4:**
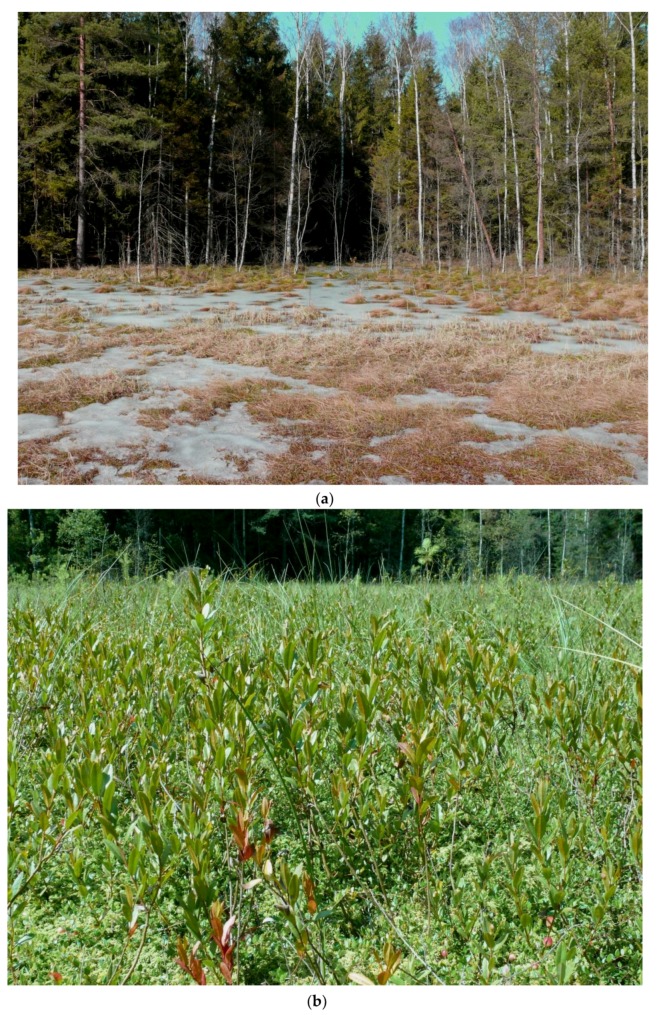
Population area of *Chamaedaphne calyculata* at the new site near Nerwik village. (**a**) Immediate surroundings (March). (**b**) Part of the studied population (August).

The research was carried out on a population of *C. calyculata* growing at a new site (Figure 1) discovered in 2010 by Dziedzic et al. [13]. Presented site is located on a mid-forest transitional bog. The hydrological system combines them with Lake Kar and with two small water bodies. In the south-eastern part of the bog there is a ditch which is occluded. As a result of melioration of the immediate vicinity, one of the water body was drained and Bagno Falaszka was created, on which sedges and willow thicket were formed. On the other two water bodies, including the Kar lake, there is a strong succession of rush reed and sedge beds. The new site is located approximately 2 km south-east of the village of Nerwik and 2.5 km north-east of the village of Giławy, at the boundary of forest sections 224f and 238a in the forest division of Wipsowo (Warmia and Mazury Province, north-eastern Poland) (Figure 2). The peat bog under discussion occupies an area of about 40 ares (4000 m^2^). The nearest environment is made of coniferous forests with more than a hundred-year-old site of common pine. The mixed forests of *Serratulo-Pinetum* (Mat., 1981) J. Mat. 1988 dominate from the northwest, and from the south-east the fresh forest *Peucedano-Pinetum* W. Mat. (1962) 1973 [6]. The banks are strongly hydrated, with open water in places. In two places the succession of *Pinus sylvestris* and *Betula pendula* is observed. The community of *C. cayculata*, covering the area of 5.5 ares (550 m^2^), is growing in the central, elevated part of the peat bog. The community is formed as a cluster of dwarf shrubs, up to 40 cm tall. The population number is a few hundred specimens (Figure 3). Most individuals bloom and fruit [13] (Figure 4).

This is a representative site, as it is one of three sites of *C. cayculata* located in the Masurian Lake District, in North-Eastern Poland, Central Europe (Figure 1). It is located near the other two sites with numerous and well-preserved populations of leatherleaf-about 15 km from site located in the Sołtysek Reserve and about 40 km from site in Piska Forest near the Krutyń village (Masurian Landscape Park).

### 2.2. Morphological Measurements of Leaves and Shoots

In March 2013, a total of 50 plants were selected at random for observation.

**Figure 5:**
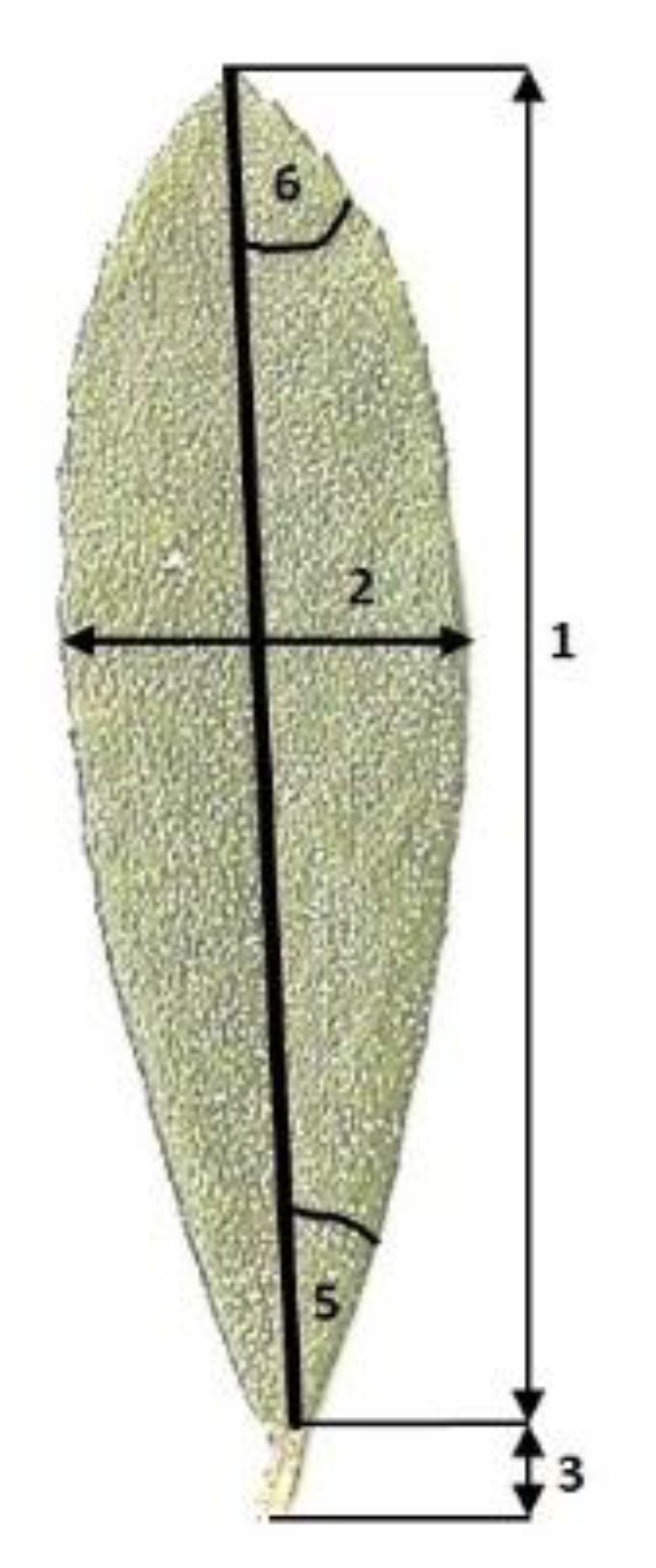
The technique of measurements of the selected parameters of *Chamaedaphne calyculata* leaves at the new site near Nerwik village (based on [7,8]).

For biometric study of morphological features of assimilating leaves six features of the leaves were considered (Figure 5): (1) length of the leaf blade, (2) width of the leaf blade, (3) length of the petiole, (4) the ratio of the length to the width of the leaf blade, (5) half of the angle at the base of the leaf blade, and (6) half of the angle at the apex of the leaf blade.

In 2013 and 2014, the examination also included such morphological features as: shoot height, maximum length of annual increments of shoots and the number of branches occurring in the examined shoots.

### 2.3. Observations of Flowering, Fruiting, and Seed Production

During the years 2013 and 2014, the number of formed flower buds, flowers and fruits was counted separately on each plant selected in March 2013. The observation of fruits was performed when they were still green and closed. To determine the number of seeds per fruit, after obtaining permits required by Polish law, a total of 50 well-developed ripe closed fruits were selected in 2014. In the laboratory, seeds were extracted, counted and used for the further experiments.

### 2.4. Germination Tests after Storage in Various Conditions

Based on the results of previous own studies (unpublished data), only mature seeds were used in the germination experiments.

The control sample was seeds stratified immediately after harvest (not stored). Experimental samples were seeds stored for 30 to 360 days in a 0–2, 2–4, 6–8, and 21–23 °C. The germination experiments were made from month 1 to months 6, 8, and 12 after harvesting. To break seed dormancy after a given storage time, the seeds were stratified on wet blotting paper at 3.5 °C for 20 days in accordance with the procedure given by Malinowska et al. [9]. Seeds were not imbibed before stratification. Seeds for a control sample, as well as experimental samples, were sown on blotting paper moistened with distilled water in 10-cm transparent glass Petri dishes. Five replicate dishes of 10 seeds each were used for each treatment. Germination tests were performed at 20 ± 2 °C in a growing apparatus using a 16-hour lighting regime. Seeds were considered germinated when their radicles protruded 2 mm. The Petri dishes were checked every two days and the germinated seeds were counted and removed from Petri dishes. Germination tests were terminated after 30 days.

In the germination tests, germination percentage (GP), time to first observed germinant (T), time to maximum germination (T_100_), mean germination time (MGT), mean germination rate (MR), germination index (GI) and germination index rate (GRI) was calculated according to formula given by Ranal et al. [26] and Nasr et al. [27].

### 2.5. Statistical Analysis

Compatibility of the distribution of the values of features with the normal distribution in the studied population was determined using the Lilliefons and Shapiro–Wilk test. In the case of studies on the dynamics of the germination process, when the distribution was inconsistent with the normal distribution, data were arcsine transformed prior to statistical analysis.

The following statistical analyses were carried out:

(1) the vegetative features and morphology of leaves: (a) to describe variables the following measures of location and variability were used: arithmetic mean (x), standard deviation (SD) and coefficient of mean variation (CV); (b) to determine the relation in the group of examined features, a Pearson rank correlation coefficient was applied. The strength of the relation was assessed taking the scale of the correlation coefficient “r”: 0 < r <0.1—dim correlation; 0.1 ≤ r <0.3—weak correlation; 0.3 ≤ r <0.5—average correlation; 0.5 ≤ r <0.7—high correlation; 0.7 ≤ r <0.9—very high correlation; 0.9 ≤ r <1.0—nearly total correlation.

(2) the generative features: (a) to describe variables the following measures of location and variability were used: arithmetic mean (x), standard deviation (SD) and coefficient of variation of mean (CV); (b) to identify significance of differences between pairs of results the sign test and Wilcoxon matched pairs test were carried out.

(3) the seed germination process: (a) to describe variables the following measures of location and variability were used: arithmetic mean (x), standard deviation (SD) and coefficient of variation of mean (CV); (b) to identify significance of differences between means from multiple independent samples the variance analysis tests were carried out; (c) to find out which specific groups of means (compared with each other) are different the results were tested by using a Tukey HSD test.

(4) comparison of the received results with literature data: (a) to determine variability of leaf morphology of the test population versus literature data the variability of morphological features of assimilating leaves based on the coefficient of variation of the mean (CV) was carried out; (b) to determine whether the reduced set of morphological features grouped the examined population and populations described in the literature in a statistically significant manner multivariate analyses (morphological distance and PCA analysis) were carried out.

In the statistical analysis of results, XLSTAT-Pro 7.5, StatSoft Statistica 13 and Microsoft Excel 2016 software were used.

## 3. Results

### 3.1. Description of the Vegetative Features and Morphology of Leaves in the Examined Population of C. calyculata

Table 1 presents descriptive statistics characterizing individual vegetative features of the shoot and morphological features of leaves in the examined population. As results from the data presented, a quite large intrapopulation morphological variability (based on coefficient of variation of mean) was observed for the observed features, ranging from 10.55% (leaf blade length/width) to about 65% (number of branches) (Table 1).

The highest variability (based on coefficient of variation of mean) was found for morphological features of leaves: leaf blade, angle at the leaf base and angle at the apex (CV ≤ 28.38%).

Within the examined population, the most variable feature (based on coefficient of variation of mean) is the length of the leaf blade (CV = 35.05%), followed by the leaf blade area (CV = 28.38%), while the least variable parameter is the leaf blade length-to-width ratio (CV = 10.55%).

A correlation analysis (based on Pearson rank correlation coefficient) revealed the existence of a series of statistically significant relations between morphological variables (Table S1). A very high positive correlation was found between the length and the area of the leaf blade, the width and the area of the leaf blade and between the length and the width of the leaf blade.

### 3.2. Description of the Generative Features in the Examined Population of C. calyculata

Intrapopulation variability of generative features of shoots (based on coefficient of variation of mean) was at the average level of 119% and amounted to, on average, 112% for the number of flower buds; 120% for the number of flowers and 126% for the number of fruit produced, respectively (Table 1).

Observations on flowering of *C. calyculata* showed abundant blooming. The number of flowers on a single specimen ranged from 0 to 60. In 2014 was almost twice as high as in 2013. On average, there were from 9 to 11 flowers per plant (Table 2).

However, it turned out that the number of fruits set from these flowers was two-fold lower. The number of fruits on one plant ranged from 0 to 25, but on average, there were from 4 to 5 fruits per plant) (Table 3).

Statistical testing the significance of differences between pairs of results (based on sign test and Wilcoxon matched pairs test) did not reveal any statistically significant differences between the values obtained for generative features of shoots received in individual years and revealed statistically significant differences between the values obtained for generative features of shoots received in a given year (Table S2, Figure S1).

On average, depending on the plant, there were 56 seeds per fruit. As regards the number of seeds in fruits, the intrapopulation variability (based on coefficient of variation of mean) was 15% (Table 1). The seeds obtained from fruits could be divided into two groups. The first was composed of developed seeds (reaching the length of about 1–1.2 mm). The other group consisted of undeveloped seeds, which were much smaller than the developed ones (about 0.25–0.4 mm). A significant difference (based on sign test and Wilcoxon matched pairs test) was observed in the number of developed and undeveloped seeds (Table S3, Figure S2). The number of seeds per fruit ranged from 31 to 48 (41 on average) for developed seeds and 10 to 20 (15 on average) for undeveloped seeds. Generally, it can be claimed that mature seeds (therefore, potentially able to germinate) were prevalent in the collected fruit.

### 3.3. The Effect of Storage Time and Temperature on Seed Germination Process in the Examined Population of C. calyculata

Table 1 presents descriptive statistics characterizing specific features of seed germination in the examined population. The intrapopulation variability (based on coefficient of variation of mean) of seed germination features was about 37% on average, and amounted to, on average, about 59% for GI; 54% for GRI; 53% for GP and 40% for T, respectively, and about 20% for other features (Table 1).

The factorial ANOVA statistical analysis demonstrated that the length of seed storage, had a significant effect on all examined parameters of the examined process (Table S4). Temperature at which the seeds were stored, apart from the T_100_ parameter, had a significant effect on the remaining germination process parameters under examination (Table S4). Interactions between the seeds’ storage time and seeds’ storage temperature factors were observed only for GP, GI, and GRI parameters (Table S4).

The correlation analysis (Table S5), based on Pearson rank correlation coefficients, demonstrated the existence of a series of statistically significant relations between the seeds’ storage time, seeds’ storage temperature and GP (a negative average correlation and a very high negative correlation, respectively), MGT (a negative weak correlation and an average positive correlation, respectively), MR (positive average correlation and negative average correlation, respectively), GI (a negative high correlation in both cases), and GRI (a negative average correlation and a very high negative correlation, respectively). The seeds storage temperature factor statistically significantly was positively correlated with the T parameter (a high correlation). A statistically significant negative correlation was observed between the T_100_ parameter and the seeds storage time factor (an average correlation) (Table S5).

**Figure 6:**
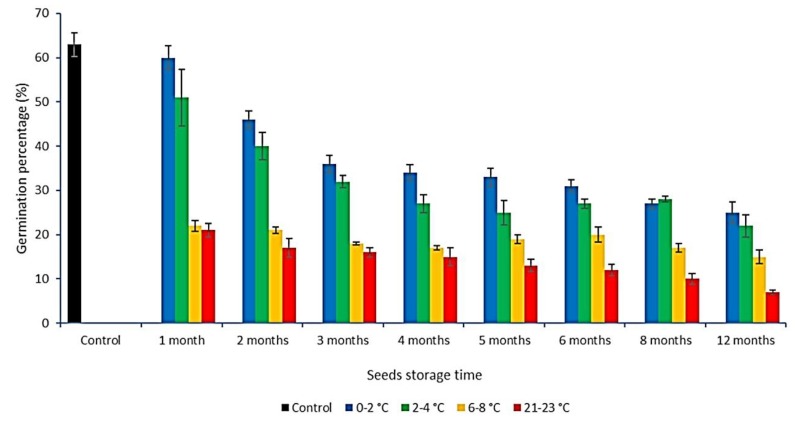
Average germination percentage of *C. calyculata* seeds stored at various temperatures for 1–6, 8, and 12 months. Vertical bars indicate a standard error (±SE).

Differences were observed in the germination of seeds subjected to different environmental conditions (Table 4, Figure 6).

As it can be read from the graph provided (Figure 6), values of the germination percentage (GP) parameter obtained for specific storage conditions were different. Statistically significant differences between the values of the GP parameter obtained in the control sample and the samples stored at 6–8 °C and room temperatures were observed after a month of seed storage. After subsequent months of storage, statistically significant differences, compared to the controls, were observed at all storage temperatures (Figure 6).

A clear decrease in the values of the germination percentage parameter was observed for seeds after subsequent months of storage at 0–2 °C and 2–4 °C—from 60% and 51% of germinating seeds after 1 month of storage up to 25% and 22% after 12 months of storage, respectively. The lowest decrease in the GP parameter was observed for seeds stored at 6–8 °C. In the sample stored at room temperature (21–23 °C), there was a twofold difference observed in the value of the GP parameter between months 1 and 12 of seed storage (Table 4). The analysis of variance and Tukey HSD test for GP parameter and seeds storage time factor revealed the existence of five homogeneous groups (Table S6A). The values obtained after first or after the second month of seed storage differed statistically significantly from all other values obtained for GP parameter. The values obtained for seeds stored for 12 months did not differ significantly only from the values obtained for seeds stored for 8 months. The analysis of the seeds storage temperature factor effect revealed the existence of four homogenous groups (Table S7A) which means, that mean values of GP parameter obtained at the tested temperatures were significantly different from each other. The analysis of the seeds storage time and temperature factors revealed the existence of 14 homogenous groups (Table S8A).

Seeds stored at room temperature, regardless of their storage time, started to germinate (T) after about 7 days, while cooled seeds started to germinate after about 5 days (storage temperature: 6–8 °C); 4 days (storage temperature: 2–4 °C). Seeds stored at 0–2 °C began to germinate the fastest, i.e., after about 3 days which correspond to time observed for control (not stored) seeds (Table 4). The analysis of variance and Tukey HSD test for T parameter and the seeds storage time factor revealed the existence of two homogenous group (Table S6B). Only the values of the T parameter obtained after 3 months and after 12 months of storage differed statistically significantly from each other. The analysis of the seeds storage temperature factor effect revealed the existence of three homogenous groups: the first group is made of means obtained for seeds stored at 0–2 °C and 2–4 °C, the second group of means obtained for seeds stored at 6–8 °C, while the third group is made of means obtained for seeds stored at room temperature (Table S7B). The analysis of the seeds storage time and temperature factors demonstrated the existence of six homogenous groups (Table S8B).

For seeds stored at room temperature and for control sample, the T_100_ parameter was on average 15 days, and for other temperatures it was 14 (Table 4). The analysis of variance and Tukey HSD test for T_100_ parameter and the seeds storage time factor revealed the existence of three homogenous groups (Table S6C). The values of T_100_ parameter obtained after one month of seed storage differed statistically significantly from values obtained after 6, 8, and 12 months of storage. The values obtained after 2 months or after 4 months of seed storage differed statistically significantly from values obtained after 8 months of storage. The analysis of seeds storage temperature factor as well as the seeds storage time and temperature factors demonstrated the existence of one homogenous group, which means that these factors did not significantly affect the values of the T_100_ parameter (Table S7C and Table S8C).

Average germination time (MGT) for control seeds was on average 9 days, for seeds stored at 0–2 °C and 2–4 °C it was 8 days, at temperature 6–8 °C it was 9 days, and for seeds stored at room temperature was on average 11 days (Table 4). The analysis of variance and Tukey HSD test for MGT parameter and the seeds storage time factor revealed the existence of two homogenous groups (Table S6D). The values obtained after fifth or after the twelfth month of seed storage did not differ statistically significantly from all other values obtained for MGT parameter. The values obtained for seeds stored for 1 and 2 months were the most different from the other. The analysis of the effect of the seeds storage temperature factor revealed the existence of two homogenous groups (Table S7D). The values of MGT parameter obtained in experiments with seeds stored at room temperature were statistically significantly different from the values obtained in experiments with seeds stored at other temperatures. There were no statistically significant differences between the values of MGT parameter obtained in the all other samples stored in the temperature range from 0 to 8°C. The analysis of the seeds storage time and temperature factors demonstrated the existence of four homogenous groups (Table S8D).

The germination rate parameter (MR) for control seeds was on average 0.11 day^−1^; for seeds stored at 0–2 °C and 2–4 °C—0.12 day^−1^; at 6–8 °C—0.11 day^−1^ and at room temperature was on average 0.09 day^−1^ (Table 4). The analysis of variance and Tukey HSD test for MR parameter and the seeds storage time factor revealed the existence of two homogenous groups (Table S6E). The values obtained after fifth or after the twelfth month of seed storage did not differ statistically significantly from all other values obtained for MGT parameter. The values obtained for seeds stored for 1 and 2 months were the most different from the other. The analysis of the effect of the seeds storage temperature factor revealed the existence of two homogenous groups (Table S7E). The values of MR parameter obtained in experiments with seeds stored at room temperature were statistically significantly different from the values obtained in experiments with seeds stored at other temperatures. There were no statistically significant differences between the values of MR parameter obtained in the all other samples stored in the temperature range from 0 to 8 °C. The analysis of the seeds storage time and temperature factors demonstrated the existence of five homogenous groups (Table S8E).

The germination index (GI) for control seeds was on average 6; for seeds stored at 0–2 °C and at 2–4 °C was on average 3; and for seeds stored at 6–8 °C and at room temperature was on average 2 (Table 4). The analysis of variance and Tukey HSD test for GI parameter and the seeds storage time factor revealed the existence of four homogenous groups (Table S6F). The values obtained after first or after the second month of seed storage differed statistically significantly from all other values obtained for GI parameter. The values of the GI parameter obtained after 3 months and after 12 months of storage differed statistically significantly also from each other. The analysis of the of the seeds storage temperature factor revealed the existence of two homogenous groups—group 1 is formed of values obtained in experiments with seeds stored at 0–2 °C and 2–4 °C, and the other is formed of values obtained in experiments with seeds stored at 6–8 °C and at room temperature (Table S7F). The analysis of the seeds storage time and temperature factors revealed the existence of six homogenous groups (Table S8F).

The germination index coefficient (GRI) for control seeds was on average 7% day^−1^; for seeds stored at 0–2 °C—5% day^−1^; at 2–4 °C—4% day^−1^; at 6–8 °C—% day^−1^ and for seeds stored at room temperature was on average 1% day^−1^ (Table 4). The analysis of variance and Tukey HSD test for GRI parameter and the seeds storage time factor demonstrated the existence of four homogenous groups (Table S6G). The values obtained after first month of seed storage differed statistically significantly from all other values obtained for GRI parameter. The values obtained for seeds stored for 12 months did not differ significantly only from the values obtained for seeds stored for 8 months. The analysis of the seeds storage temperature factor effect revealed the existence of four homogenous groups (Table S7G) which means, that mean values of GRI parameter obtained at the tested temperatures were significantly different from each other. The analysis of the seeds storage time and temperature factors revealed the existence of 14 homogenous groups (Table S8G).

## 4. Discussion

A comparison of the variability of morphological features in the examined population with the features of the population described by Polakowski [5], Klimko et al. [24], and Klimko and Szkudlarz [25] demonstrated that the examined population was characterized by the highest variability of the petiole length among all populations. The leaves of specimens growing at the newly discovered site were distinguished by the highest values of angles at the apex of the leaf blade and had the second largest (just after the population originating from the reserve “Sieraków” in the Kampinos National Park) leaf base angles. Additionally, the examined population, together with the population growing in Sowiniec in the Bory Tucholskie, was one of the two most variable populations out of all described populations in the literature [24,25] in terms of leaf morphology. With regard to other features, the examined population revealed variability corresponding to the literature data provided for other populations (Table S9). The analysis of the morphological distance calculated on the basis of the Euclidean distance between populations, using six morphological features of leaves, demonstrated that individual populations are generally characterized by small morphological differences between them. The average distance for all populations was 11.62. The largest distance was found between the population examined in this study and the population originating from the Sitno site (Table S10). Despite this, it can be clearly seen that the populations are similar in morphological terms. As results from the analysis of the variation coefficient, relatively high intrapopulation variability is observed in the examined population. A principal component analysis (PCA) carried out on the basis of the covariance matrix, created from converting the values for six features of leaves, demonstrated that the examined population reveals a high morphological difference when compared to the populations examined by Klimko et al. [24] and Klimko and Szkudlarz [25], and it forms a separate group (Figure S3).

Observations carried out by Tylżanowski [28] and Malinowska et al [9], at sites in the Bory Tucholskie Forests and “Sicienko” nature reserve in the Drawa National Park, respectively, showed that leather leaf flowers abundantly but does not set many fruits (about 10% of flowers were transformed into fruits). Similarly, Wróblewska [3] reported that in two of the nine marginal populations in Central Europe (Jesionowe Góry Reserve and Kampinos National Park, Poland), flowering and fruit set have been sporadic or absent over several years. Research conducted by Reader [29] indicates that the American population show a high frequency of seed set (50–95%) when flowers were open-pollinated, and a low seed set (1–15%) when flowers were self-fertilized. On the other hand, Les [30], describing *C. calyculata* occurring in North America, indicates that flowers are with high seed set, occurring regardless of whether self-pollination or open-pollination took place. In the current study, about 50% flowers were observed transformed into fruit, which corresponded to the data presented by Les [30]. Our laboratory investigations showed that many seeds were found in fruits. The number of seeds observed (on average 56 per fruit), was similar to the number of seeds observed by Malinowska et al. [9] for the population from Poland—on average 65 seeds per fruit and by Les [30] for a population from North America—on average 60 seeds were produced by each capsule. However, contrary to the observation made by Malinowska et al. [9], in the current study the percentage of developed seeds was very high (about 73%). The work of Malinowska et al. [9] also signaled the interesting phenomenon of producing morphologically diversified seeds in one capsule, which were described as flat and convex. It was similar in our case—we distinguished large, ripe seeds as well as small, immature seeds. The production of the hetromorphic diasporas was found in 18 families of angiosperm plants (99 species, 218 species) [31], however no work on the heteromorphism of any representative of Ericaceae is known. In the future, it would be necessary to delve into this aspect of reproduction, get to know the possible impact of environmental conditions on the production of dimorphic seeds, and then the differentiation of the response of seeds to specific germination conditions.

It should be emphasized that there are currently no literature data concerning detailed and extensive research on the germination process of leatherleaf (a representative of the Ericaceae family, the only representative of the *Chamaedaphne* genus). Laboratory seed germination tests carried out by the authors using various temperatures and times of stratification treatment allowed the effect of those factors to be observed on various germination parameters (Table 4). Data presented by Desmore [10], Les [30], and Deno [32,33,34] indicate that germination of *C. calyculata* seeds requires light, which was also observed in previous pilot experiments (data not shown). Both the literature data [9,10,30,32,33,34,35] and the results of our previous research allow us to conclude that non-stratified seeds of leatherleaf demonstrate poor germination (at the level of 20%), which was comparable to the results achieved for seeds stored at room temperature. This fact proves the occurrence of seeds with physiological dormancy [35]. Similar to a study by Malinowska et al. [9], it was observed that a higher seed germination index was found for seeds stored at lower temperatures. Malinowska et al. [9] observed that *C. calyculata* seeds germinate up to 10 days and Deno [34] reports that the germination process lasts from day 9 to 17 of the experiment and after 6 and 12 months of seed storage it lasts for four weeks. In the current study, the T_100_ parameter averaged 15 days for seeds stored at room temperature and 14 days for other temperatures. The results of the current study and the quoted studies concerning the process of leather leaf seed germination indicate that not only vegetative, but also generative propagation is possible in this species. Deno [34] also reported that the half-life of *C. calyculata* seeds is 6 months, while Les [30] claims that this species does not create a seed bank. Those statements are consistent with the results of the current study.

## 5. Conclusions

Presented results describe the population of *Chamaedaphne calyculata* (L.) Moench growing at a newly discovered site located in North-Eastern Poland (Central Europe) in a comprehensive manner. Thus, it is the first detailed report on the morphological features of shoots and morphometrics of leaves, generative features (flowering, fruiting and seed production) and parameters of the *C. calyculata* seed germination process, which have not been presented before.

The ability of seeds to germinate (and consequently, the generative propagation of the leather leaf), non-creation of the seed bank and other results of the research provide new information necessary for planning and carrying out protective procedures (including active conservation of plants in ex situ conditions).

It should be emphasized that biology of *C. calyculata* is still poorly understood. Therefore, in our further research we will look for the answer to the questions: what factors limit generative reproduction of this species in natural conditions; if and why seeds do not germinate under natural conditions and what are the reasons for setting up such a small number of seeds.

## Figures and Tables

**Table 1:** The basic statistics of quantitative variables of vegetative features of the shoots; morphological features of leaves; generative features of shoot and features of seed germination process in the examined *C. calyculata* population. Abbreviations: arithmetic mean (x), standard deviation (SD), coefficient of mean variation (CV), germination percentage (GP), time to first observed germinant (T), time to maximum germination (T_100_), mean germination time (MGT), mean germination rate (MR), germination index (GI), and germination index rate (GRI)

		Average	Min.	Max.	SD	CV	Average CV
**Vegetative features of shoot**	Height (cm)	55.64	21.00	97.00	18.30	32.89%	62.68%
	Maximum length of increments	13.13	0.00	33.50	8.03	61.14%	
	Average length of increments	9.42	0.00	19.00	4.87	51.72%	
	Number of branches	3.36	0.00	9.00	2.18	64.97%	
**Leaf morphological features**	Length of leaf blade	29.79	18.33	42.12	4.99	16.74%	20.51%
	Leaf blade width	10.13	6.74	12.93	1.43	14.10%	
	Length of petiole	1.75	0.44	2.80	0.61	35.05%	
	Leaf blade length-to-width ratio	2.94	2.37	3.66	0.31	10.55%	
	Half of the angle at the leaf blade base	48.72	25.27	69.27	9.32	19.14%	
	Half of the angle at the leaf blade apex	61.20	29.58	88.12	12.01	19.63%	
	Leaf blade area (mm^2^)	231.25	101.13	369.00	65.64	28.38%	
**Generative features of shoot**	Number of flower buds	13.72	0.00	61.00	15.33	111.73%	119.55%
	Number of flowers	9.93	0.00	60.00	11.97	120.57%	
	Number of produced fruits	4.72	0.00	25.00	5.96	126.37%	
**Seeds**	Number of seeds in fruit	59.90	43.00	68	8.49	15.19%	15.19%
**Features of the seed germination process parameters**	GP	26.34	6.00	70.00	13.93	52.87%	37.23%
	T	4.69	2.00	12.00	1.77	38.00%	
	T_100_	14.08	8.00	21.00	2.62	18.58%	
	MGT	9.17	6.12	15.43	1.81	19.79%	
	MR	0.11	0.06	0.16	0.02	18.57%	
	GI	2.40	0.50	7.34	1.41	58.79%	
	GRI	3.53	0.45	7.76	1.90	54.00%	

**Table 2:** Characteristics of flowering of *C. calyculata* in the examined population in subsequent years. Values with ± SE are means.

Year	Total Number of Flower Buds	Min. and Max. Number of Flower Buds on a Single Plant	Average Number of flower Buds on a Single Plant	Total Number of Formed Flowers	Min. and Max. Number of Formed Flowers on a Single Plant	Average Number of Formed Flowers on a Single Plant
2013	669	0–61	13 ± 1.98	449	0–34	9 ± 1.37
2014	703	0–61	14 ± 2.39	544	0–60	11 ± 2.02
Average for total	686	0–61	14 ± 1.53	496	0–47	10 ± 1.20

**Table 3:** Characteristics of fruit forming of *C. calyculata* in the examined population in subsequent years. Values with ± SE are means.

Year	Total Number of Formed Fruits	Min. and Max. Number of Formed Fruits on a Single Plant	Average Number of Formed Fruits on a Single Plant
2013	203	0–17	4 ± 0.70
2014	269	0–25	5 ± 0.98
Average for total	236	0–21	5 ± 0.60

**Table 4:** The germination characteristics of *C. calyculata* seeds stored in various temperatures for various periods of time. The results are the means with standard error (±SE). The numbers followed by the same letter in each column at a given seeds storage temperature are not significantly different (*p* < 0.05).

Seeds Storage Temp. (°C)	Seeds Storage Time (months)	T (day)	T_100_ (day)	MGT (day)	MR (day ^−1^)	GI	GRI (% day ^−1^)
control	0	3 ± 0.24	15 ± 0.40	9.40 ± 0.31	0.11 ± 0.00	5.96 ± 0.40	7.40 ± 0.11
0–2							
	1	3 ± 0.24ab	16 ± 0.60a	9.75 ± 0.28a	0.10 ± 0.01ab	5.87 ± 0.40a	7.40 ± 0.11a
	2	3 ± 0.24ab	15 ± 0.49a	10.23 ± 0.55a	0.10 ± 0.01a	4.69 ± 0.43a	5.64 ± 0.22bc
	3	3 ± 0.40a	14 ± 0.95a	7.73 ± 0.48b	0.13 ± 0.01bc	2.78 ± 0.22b	6.26 ± 0.61ab
	4	3 ± 0.24ab	13 ± 1.32a	7.74 ± 0.58b	0.13 ± 0.01c	2.66 ± 0.31b	5.40 ± 0.32bcd
	5	4 ± 0.20ab	14 ± 0.97a	8.38 ± 0.40ab	0.12 ± 0.01abc	2.80 ± 0.28b	4.73 ± 0.09cde
	6	4 ± 0.24ab	12 ± 1.58a	7.39 ± 0.19b	0.14 ± 0.00c	2.27 ± 0.10b	4.91 ± 0.20bcde
	8	4 ± 0.37b	12 ± 0.92a	7.49 ± 0.13b	0.13 ± 0.00c	2.04 ± 0.10b	4.14 ± 0.18de
	12	4 ± 0.32ab	13 ± 1.24a	7.78 ± 0.50b	0.13 ± 0.01bc	1.94 ± 0.27b	3.81 ± 0.42e
2–4							
	1	4 ± 0.49ab	15 ± 1.60a	9.61 ± 0.73a	0.11 ± 0.01a	5.05 ± 0.84a	6.19 ± 0.59a
	2	4 ± 0.24ab	14 ± 1.30a	9.56 ± 0.47a	0.11 ± 0.01a	3.91 ± 0.43ab	5.13 ± 0.41ab
	3	3 ± 0.40a	15 ± 0.63a	8.64 ± 0.40a	0.12 ± 0.01a	2.77 ± 0.19bc	4.65 ± 0.38abc
	4	4 ± 0.24ab	15 ± 0.86a	8.02 ± 0.47a	0.13 ± 0.01a	2.18 ± 0.29bc	4.14 ± 0.33bc
	5	4 ± 0.32ab	14 ± 1.40a	8.27 ± 0.56a	0.12 ± 0.01a	2.09 ± 0.28bc	3.67 ± 0.50bc
	6	4 ± 0.93ab	13 ± 1.29a	8.35 ± 0.91a	0.12 ± 0.01a	2.28 ± 0.28bc	4.00 ± 0.42bc
	8	4 ± 0.32ab	12 ± 1.03a	7.46 ± 0.15a	0.13 ± 0.00a	2.09 ± 0.06bc	4.27 ± 0.09bc
	12	5 ± 0.86b	13 ± 1.05a	8.48 ± 0.69a	0.12 ± 0.01a	1.95 ± 0.34c	3.09 ± 0.39c
6–8							
	1	5 ± 0.20a	16 ± 1.20a	10.19 ± 0.88a	0.10 ± 0.01a	2.21 ± 0.24a	2.52 ± 0.25ab
	2	5 ± 0.86a	15 ± 0.60a	10.15 ± 0.48a	0.10 ± 0.01a	2.14 ± 0.09a	2.48 ± 0.26ab
	3	5 ± 0.37a	13 ± 0.87a	8.54 ± 0.27a	0.12 ± 0.00a	1.57 ± 0.06ab	2.46 ± 0.14ab
	4	4 ± 0.37a	14 ± 1.16a	8.28 ± 0.45a	0.12 ± 0.01a	1.43 ± 0.10b	2.55 ± 0.19ab
	5	4 ± 0.24a	14 ± 1.10a	8.84 ± 0.56a	0.11 ± 0.01a	1.71 ± 0.17ab	2.55 ± 0.18ab
	6	4 ± 0.55a	12 ± 1.07a	7.71 ± 0.33a	0.13 ± 0.01a	1.54 ± 0.15ab	3.17 ± 0.36b
	8	5 ± 0.37a	12 ± 0.86a	8.34 ± 0.35a	0.12 ± 0.00a	1.43 ± 0.09b	2.32 ± 0.17ab
	12	5 ± 0.73a	13 ± 0.95a	8.86 ± 0.35a	0.12 ± 0.01a	1.34 ± 0.18b	1.99 ± 0.24a
21–23							
	1	7 ± 0.55a	17 ± 0.20a	11.35 ± 0.36a	0.09 ± 0.00a	2.35 ± 0.14a	2.07 ± 0.24a
	2	8 ± 1.20a	15 ± 0.37a	11.12 ± 0.62a	0.09 ± 0.00a	1.87 ± 0.17ab	1.73 ± 0.32ab
	3	6 ± 0.37a	14 ± 0.95a	9.80 ± 0.82a	0.10 ± 0.01a	1.57 ± 0.16abc	1.88 ± 0.22ab
	4	6 ± 0.75a	16 ± 1.61a	10.93 ± 1.02a	0.09 ± 0.01a	1.60 ± 0.25abc	1.58 ± 0.30abc
	5	7 ± 0.73a	16 ± 1.17a	11.92 ± 0.99a	0.09 ± 0.01a	1.54 ± 0.21abc	1.19 ± 0.18abc
	6	6 ± 0.75a	14 ± 1.96a	10.40 ± 1.30a	0.10 ± 0.01a	1.35 ± 0.29bc	1.31 ± 0.09abc
	8	6 ± 0.51a	13 ± 1.46a	10.19 ± 0.90a	0.10 ± 0.01a	1.09 ± 0.20bc	1.10 ± 0.10bc
	12	7 ± 1.41a	14 ± 1.24a	11.42 ± 0.96a	0.09 ± 0.01a	0.78 ± 0.10c	0.63 ± 0.05c

## Supplementary Materials

The following are available online at https://www.mdpi.com/2223-7747/8/5/129/s1, Figure S1: Plot (box and whisker) presenting the result of testing the significance of differences between pairs of results for generative features of *C. calyculata* shoots in the examined population, Figure S2: Plot (box and whisker) presenting the result of testing the significance of differences between pairs of results for seeds of *C. calyculata* in the examined population, Figure S3: Principal component analysis (PCA) of the examined population of *C. calyculata* (“New population”) and literature data [5,26,27] based on morphological features, Table S1: Pearson rank correlation coefficients between tested leaf morphological features of *C. calyculata* in the examined population. Statistically significant coefficients (*p* ≤ 0.05) are in bold on gray background, Table S2: The significance of differences between pairs of results for generative features of *C. calyculata* shoots in the examined population. Statistically significant tests (*p* < 0.05) are in bold on gray background, Table S3: The significance of differences between pairs of results for seeds of *C. calyculata* in the examined population. Statistically significant tests (*p* < 0.05) are in bold on gray background, Table S4: Factorial (seeds storage time and seeds storage temperature) ANOVA test for parameters of the dynamics of seed germination in the examined *C. calyculata* population. Univariate results for each germinations parameter. Sigma-restricted parameterization. Effective hypothesis decomposition. Statistically significant results (α = 0.05) are in bold on gray background, Table S5: Pearson rank correlation coefficients between tested parameters of the dynamics of seed germination and seeds storage time and temperature in the examined *C. calyculata* population. Statistically significant coefficients (*p* ≤ 0.05) are in bold on gray background, Table S6: Homogenous groups of means of the seed germination dynamics parameters determined on the basis of the Tukey HSD test (α = 0.05) with seeds storage time factor, Table S7: Homogenous groups of means of the seed germination dynamics parameters determined on the basis of the Tukey HSD test (α = 0.05) with seeds storage temperature factor, Table S8: Homogenous groups of means of the seed germination dynamics parameters determined on the basis of the Tukey HSD test (α = 0.05) with two factors: seeds storage time and seeds storage temperature, Table S9: Variability of morphological features of assimilating leaves leaf of the examined population of *C. calyculata* (“New population”) versus literature data [5,24,25] based on the coefficient of variation of the mean (CV), Table S10: Morphological distance between the examined population of *C. calyculata* (“New population”) versus literature data [5,26,27] based on Euclidean distance using 6 morphological features of leaves.
