# Supplementary material for: The Morphological Features and Biology of a Relict and Endangered Woody Plant Species: Chamaedaphne calyculata (L.) Moench (Ericaceae)
Source: Plants (Basel). 2019 May 15;8(5):129. doi: 10.3390/plants8050129 (PMC6572642; doi:10.3390/plants8050129)
Supplement: Supplementary file 1 [file plants-08-00129-s001.zip › Fig. S2.docx]

**Figure S2.** Plot (box and whisker) presenting the result of testing the significance of differences between pairs of results for seeds of *C. calyculata* in the examined population.
