# Supplementary material for: The Morphological Features and Biology of a Relict and Endangered Woody Plant Species: Chamaedaphne calyculata (L.) Moench (Ericaceae)
Source: Plants (Basel). 2019 May 15;8(5):129. doi: 10.3390/plants8050129 (PMC6572642; doi:10.3390/plants8050129)
Supplement: Supplementary file 1 [file plants-08-00129-s001.zip › Fig. S3.docx]

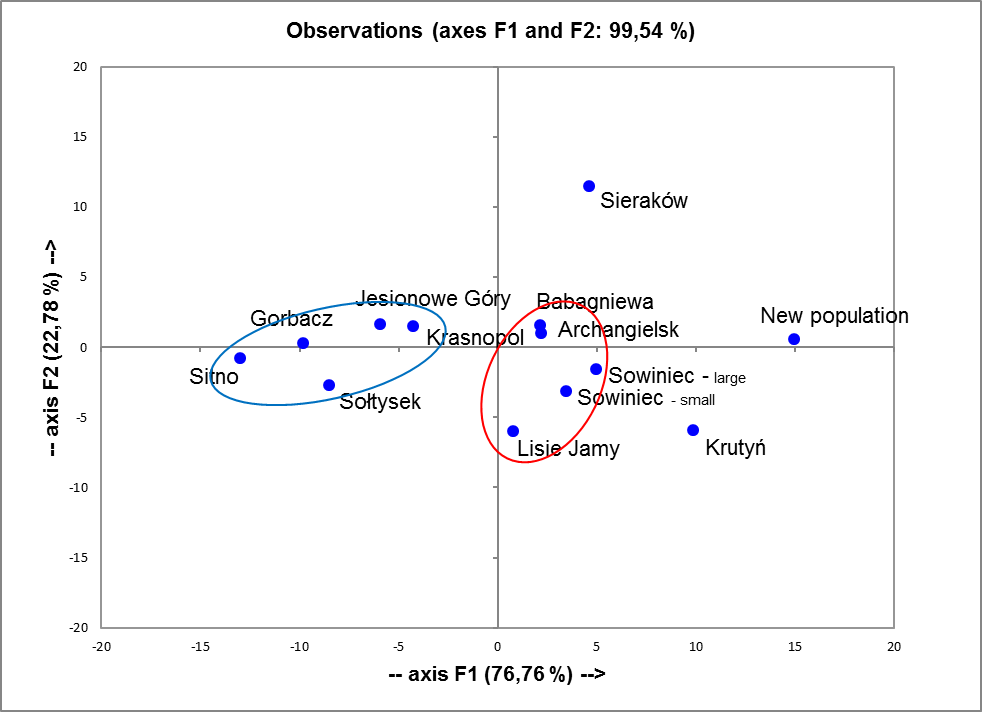


Figure S3. Principal component analysis (PCA) of the examined population of *Ch. calyculata* (“New population”) and literature data [5, 24, 25] based on morphological features.
