# Supplementary material for: The Morphological Features and Biology of a Relict and Endangered Woody Plant Species: Chamaedaphne calyculata (L.) Moench (Ericaceae)
Source: Plants (Basel). 2019 May 15;8(5):129. doi: 10.3390/plants8050129 (PMC6572642; doi:10.3390/plants8050129)
Supplement: Supplementary file 1 [file plants-08-00129-s001.zip › Table S1.docx]

**Table S1.** Pearson rank correlation coefficients between tested leaf morphological features of *C. calyculata* in the examined population. Statistically significant coefficients (p ≤ 0,05) are in bold on gray background.

|  | **Length of leaf blade** | **Length of petiole** | **Width of leaf blade** | **Half of the angle at the leaf blade base** | **Half of the angle at the leaf blade apex** | **Leaf blade area (mm^2^)** |
| --- | --- | --- | --- | --- | --- | --- |
| **Length of leaf blade** | 1.00 |  |  |  |  |  |
| **Length of petiole** | **0.39** | 1.00 |  |  |  |  |
| **Width of leaf blade** | **0.79** | **0.36** | 1.00 |  |  |  |
| **Half of the angle at the leaf blade base** | -0.22 | -0.10 | 0.08 | 1.00 |  |  |
| **Half of the angle at the leaf blade apex** | -0.28 | -0.24 | -0.13 | **0.33** | 1.00 |  |
| **Leaf blade area (mm^2^)** | **0.86** | **0.40** | **0.84** | -0.12 | -0.26 | 1.00 |
