# Supplementary material for: The Morphological Features and Biology of a Relict and Endangered Woody Plant Species: Chamaedaphne calyculata (L.) Moench (Ericaceae)
Source: Plants (Basel). 2019 May 15;8(5):129. doi: 10.3390/plants8050129 (PMC6572642; doi:10.3390/plants8050129)
Supplement: Supplementary file 1 [file plants-08-00129-s001.zip › Table S10.docx]

Table S10. Morphological distance between the examined population of *Ch. calyculata* (“New population”) versus literature data [5, 24, 25] based on Euclidean distance using 6 morphological features of leaves.

|  | **Krasnopol** | **Jesionowe Góry** | **Gorbacz** | **Lisie Jamy** | **Krutyń** | **Sołtysek** | **Sieraków** | **Sowiniec - small leaf blade** | **Sowiniec - large leaf blade** | **Sitno** | **Babagniewa** | **Archangielsk** | **New population** |
| --- | --- | --- | --- | --- | --- | --- | --- | --- | --- | --- | --- | --- | --- |
| **Krasnopol** | 0.0 |  |  |  |  |  |  |  |  |  |  |  |  |
| **Jesionowe Góry** | 1.7 | 0.0 |  |  |  |  |  |  |  |  |  |  |  |
| **Gorbacz** | 5.8 | 4.4 | 0.0 |  |  |  |  |  |  |  |  |  |  |
| **Lisie Jamy** | 9.0 | 10.2 | 12.3 | 0.0 |  |  |  |  |  |  |  |  |  |
| **Krutyn** | 15.9 | 17.5 | 20.6 | 9.1 | 0.0 |  |  |  |  |  |  |  |  |
| **Sołtysek** | 6.0 | 5.1 | 3.4 | 9.9 | 18.7 | 0.0 |  |  |  |  |  |  |  |
| **Sieraków** | 13.3 | 14.4 | 18.3 | 17.9 | 18.1 | 19.3 | 0.0 |  |  |  |  |  |  |
| **Sowiniec - small leaf blade** | 9.0 | 10.6 | 13.7 | 4.0 | 7.0 | 12.0 | 14.7 | 0.0 |  |  |  |  |  |
| **Sowiniec - large leaf blade** | 9.8 | 11.4 | 15.0 | 6.2 | 6.6 | 13.6 | 13.1 | 2.7 | 0.0 |  |  |  |  |
| **Sitno** | 9.0 | 7.5 | 3.5 | 14.7 | 23.4 | 4.9 | 21.4 | 16.6 | 18.0 | 0.0 |  |  |  |
| **Babagniewa** | 6.4 | 8.1 | 12.0 | 7.7 | 10.8 | 11.5 | 10.2 | 4.9 | 4.4 | 15.3 | 0.0 |  |  |
| **Archangielsk** | 6.6 | 8.3 | 12.0 | 7.2 | 10.4 | 11.4 | 10.7 | 4.4 | 4.3 | 15.3 | 1.2 | 0.0 |  |
| **New population** | 19.2 | 20.9 | 24.8 | 15.7 | 8.3 | 23.7 | 15.0 | 12.2 | 10.2 | 28.0 | 12.9 | 12.8 | 0.0 |
