# Supplementary material for: The Morphological Features and Biology of a Relict and Endangered Woody Plant Species: Chamaedaphne calyculata (L.) Moench (Ericaceae)
Source: Plants (Basel). 2019 May 15;8(5):129. doi: 10.3390/plants8050129 (PMC6572642; doi:10.3390/plants8050129)
Supplement: Supplementary file 1 [file plants-08-00129-s001.zip › Table S2.docx]

**Table S2.** The significance of differences between pairs of results for generative features of *C. calyculata* shoots in the examined population. Statistically significant tests (p < 0.05) are in bold on gray background.

|  | **Sign Test** | | | |
| --- | --- | --- | --- | --- |
|  | \| **Z** \| \| --- \| | | \| **p-value** \| \| --- \| | |
| \| Number of flower buds in 2013 & Number of flowers in 2013 \| \| --- \| | **5.39** | | **0.00** | |
| \| Number of flower buds in 2013 & Number of fruits in 2013 \| \| --- \| | **5.92** | | **0.00** | |
| \| Number of flowers in 2013 & Number of fruits in 2013 \| \| --- \| | **5.48** | | **0.00** | |
| \| Number of flower buds in 2014 & Number of flowers in 2014 \| \| --- \| | **5.00** | | **0.00** | |
| \| Number of flower buds in 2014 & Number of fruits in 2014 \| \| --- \| | **5.29** | | **0.00** | |
| \| Number of flowers in 2014 & Number of fruits in 2014 \| \| --- \| | **5.10** | | **0.00** | |
| \| Number of flower buds in 2013 & Number of flower buds in 2014 \| \| --- \| | 0.45 | | 0.65 | |
| \| Number of flowers in 2013 & Number of flowers in 2014 \| \| --- \| | 0.15 | | 0.88 | |
| \| Number of fruits in 2013 & Number of fruits in 2014 \| \| --- \| | 0.96 | | 0.34 | |
|  | **Wilcoxon Matched Pairs Test** | | | |
|  | \| **T** \| \| --- \| | \| **Z** \| \| --- \| | | \| **p-value** \| \| --- \| |
| \| Number of flower buds in 2013 & Number of flowers in 2013 \| \| --- \| | **0.00** | **4.86** | | **0.00** |
| \| Number of flower buds in 2013 & Number of fruits in 2013 \| \| --- \| | **0.00** | **5.30** | | **0.00** |
| \| Number of flowers in 2013 & Number of fruits in 2013 \| \| --- \| | **0.00** | **4.94** | | **0.00** |
| \| Number of flower buds in 2014 & Number of flowers in 2014 \| \| --- \| | **0.00** | **4.54** | | **0.00** |
| \| Number of flower buds in 2014 & Number of fruits in 2014 \| \| --- \| | **0.00** | **4.78** | | **0.00** |
| \| Number of flowers in 2014 & Number of fruits in 2014 \| \| --- \| | **0.00** | **4.62** | | **0.00** |
| \| Number of flower buds in 2013 & Number of flower buds in 2014 \| \| --- \| | 488.00 | 0.08 | | 0.93 |
| \| Number of flowers in 2013 & Number of flowers in 2014 \| \| --- \| | 438.00 | 0.66 | | 0.50 |
| \| Number of fruits in 2013 & Number of fruits in 2014 \| \| --- \| | 302.50 | 1.22 | | 0.22 |
