# Supplementary material for: The Morphological Features and Biology of a Relict and Endangered Woody Plant Species: Chamaedaphne calyculata (L.) Moench (Ericaceae)
Source: Plants (Basel). 2019 May 15;8(5):129. doi: 10.3390/plants8050129 (PMC6572642; doi:10.3390/plants8050129)
Supplement: Supplementary file 1 [file plants-08-00129-s001.zip › Table S3.docx]

**Table S3.** The significance of differences between pairs of results for seeds of *C. calyculata* in the examined population. Statistically significant tests (p < 0.05) are in bold on gray background.

|  | **Sign Test** | | | |
| --- | --- | --- | --- | --- |
|  | \| **Z** \| \| --- \| | | \| **p-value** \| \| --- \| | |
| \| Developed seeds & Undeveloped seeds \| \| --- \| | **2.85** | | **0.00** | |
|  | **Wilcoxon Matched Pairs Test** | | | |
|  | \| **T** \| \| --- \| | \| **Z** \| \| --- \| | | \| **p-value** \| \| --- \| |
| \| Developed seeds & Undeveloped seeds \| \| --- \| | **0.00** | **2.80** | | **0.01** |
