# Supplementary material for: The Morphological Features and Biology of a Relict and Endangered Woody Plant Species: Chamaedaphne calyculata (L.) Moench (Ericaceae)
Source: Plants (Basel). 2019 May 15;8(5):129. doi: 10.3390/plants8050129 (PMC6572642; doi:10.3390/plants8050129)
Supplement: Supplementary file 1 [file plants-08-00129-s001.zip › Table S4.docx]

**Table S4.** Factorial (seeds storage time and seeds storage temperature) ANOVA test for parameters of the dynamics of seed germination in the examined *C. calyculata* population. Univariate results for each germinations parameter. Sigma-restricted parameterization. Effective hypothesis decomposition. Statistically significant results (α=0.05) are in bold on gray background.

|  | \| **GP F** \| \| --- \| | \| **GP p** \| \| --- \| | \| **T F** \| \| --- \| | \| **T p** \| \| --- \| | \| **T_100_ F** \| \| --- \| | \| **T_100_ p** \| \| --- \| | \| **MGT F** \| \| --- \| | \| **MGT p** \| \| --- \| | \| **MR F** \| \| --- \| | \| **MR p** \| \| --- \| | \| **GI F** \| \| --- \| | \| **GI p** \| \| --- \| | \| **GRI F** \| \| --- \| | \| **GRI p** \| \| --- \| |
| --- | --- | --- | --- | --- | --- | --- | --- | --- | --- | --- | --- | --- | --- | --- | --- | --- | --- | --- | --- | --- | --- | --- | --- | --- | --- | --- | --- | --- |
| \| **Seeds storage time** \| \| --- \| | **43.70** | **0.00** | **2.20** | **0.00** | **5.30** | **0.00** | **5.40** | **0.00** | **6.40** | **0.00** | **34.30** | **0.00** | **18.60** | **0.00** |
| \| **Seeds storage temperature** \| \| --- \| | **222.50** | **0.00** | **53.70** | **0.00** | 1.70 | 0.20 | **27.00** | **0.00** | **23.30** | **0.00** | **67.10** | **0.00** | **267.80** | **0.00** |
| \| **Seeds storage time * seeds storage temperature** \| \| --- \| | **5.60** | **0.00** | 0.60 | 0.90 | 0.30 | 1.00 | 0.60 | 0.90 | 0.60 | 0.90 | **3.90** | **0.00** | **3.20** | **0.00** |
