# Supplementary material for: The Morphological Features and Biology of a Relict and Endangered Woody Plant Species: Chamaedaphne calyculata (L.) Moench (Ericaceae)
Source: Plants (Basel). 2019 May 15;8(5):129. doi: 10.3390/plants8050129 (PMC6572642; doi:10.3390/plants8050129)
Supplement: Supplementary file 1 [file plants-08-00129-s001.zip › Table S5.docx]

**Table S5.** Pearson rank correlation coefficients between tested parameters of the dynamics of seed germination and seeds storage time and temperature in the examined *C. calyculata* population. Statistically significant coefficients (p ≤ 0,05) are in bold on gray background.

|  | \| **Seeds storage**  **time** \| \| --- \| | \| **Seeds storage**  **temperature** \| \| --- \| |
| --- | --- | --- | --- | --- |
| \| **GP** \| \| --- \| | **-0.46** | **-0.70** |
| \| **T** \| \| --- \| | **0.06** | **0.61** |
| \| **T_100_** \| \| --- \| | **-0.40** | **0.11** |
| \| **MGT** \| \| --- \| | **-0.26** | **0.45** |
| \| **MR** \| \| --- \| | **0.30** | **-0.44** |
| \| **GI** \| \| --- \| | **-0.54** | **-0.52** |
| \| **GRI** \| \| --- \| | **-0.32** | **-0.81** |
