# Supplementary material for: The Morphological Features and Biology of a Relict and Endangered Woody Plant Species: Chamaedaphne calyculata (L.) Moench (Ericaceae)
Source: Plants (Basel). 2019 May 15;8(5):129. doi: 10.3390/plants8050129 (PMC6572642; doi:10.3390/plants8050129)
Supplement: Supplementary file 1 [file plants-08-00129-s001.zip › Table S6.docx]

**Table S6**. Homogenous groups of means of the seed germination dynamics parameters determined on the basis of the Tukey HSD test (α = 0.05) with seeds storage time factor.

A. Germination percentage (GP)

| \| **Seeds storage time**  **(months)** \| \| --- \| | \| **1** \| \| --- \| | \| **2** \| \| --- \| | \| **3** \| \| --- \| | \| **4** \| \| --- \| | \| **5** \| \| --- \| |
| --- | --- | --- | --- | --- | --- | --- | --- | --- | --- | --- | --- |
| 1 | **** |  |  |  |  |
| 2 |  | **** |  |  |  |
| 3 |  |  | **** |  |  |
| 4 |  |  | **** | **** |  |
| 5 |  |  | **** | **** |  |
| 6 |  |  | **** | **** |  |
| 8 |  |  |  | **** | **** |
| 12 |  |  |  |  | **** |

B. Time to first observed germinant (T)

| \| **Seeds storage time**  **(months)** \| \| --- \| | \| **1** \| \| --- \| | \| **2** \| \| --- \| |
| --- | --- | --- | --- | --- | --- |
| 1 | **** | **** |
| 2 | **** | **** |
| 3 | **** |  |
| 4 | **** | **** |
| 5 | **** | **** |
| 6 | **** | **** |
| 8 | **** | **** |
| 12 |  | **** |

C. Time to maximum germination (T_100_)

| \| **Seeds storage time**  **(months)** \| \| --- \| | \| **1** \| \| --- \| | \| **2** \| \| --- \| | \| **3** \| \| --- \| |
| --- | --- | --- | --- | --- | --- | --- | --- |
| 1 | **** |  |  |
| 2 | **** | **** |  |
| 3 | **** | **** | **** |
| 4 | **** | **** |  |
| 5 | **** | **** | **** |
| 6 |  | **** | **** |
| 8 |  |  | **** |
| 12 |  | **** | **** |

D. Mean germination time (MGT)

| \| **Seeds storage time**  **(months)** \| \| --- \| | \| **1** \| \| --- \| | \| **2** \| \| --- \| |
| --- | --- | --- | --- | --- | --- |
| 1 | **** |  |
| 2 | **** |  |
| 3 |  | **** |
| 4 |  | **** |
| 5 | **** | **** |
| 6 |  | **** |
| 8 |  | **** |
| 12 | **** | **** |

E. Mean germination rate (MR)

| \| **Seeds storage time**  **(months)** \| \| --- \| | \| **1** \| \| --- \| | \| **2** \| \| --- \| |
| --- | --- | --- | --- | --- | --- |
| 1 | **** |  |
| 2 | **** |  |
| 3 |  | **** |
| 4 |  | **** |
| 5 | **** | **** |
| 6 |  | **** |
| 8 |  | **** |
| 12 | **** | **** |

F. Germination index (GI)

| \| **Seeds storage time**  **(months)** \| \| --- \| | \| **1** \| \| --- \| | \| **2** \| \| --- \| | \| **3** \| \| --- \| | \| **4** \| \| --- \| |
| --- | --- | --- | --- | --- | --- | --- | --- | --- | --- |
| 1 | **** |  |  |  |
| 2 |  | **** |  |  |
| 3 |  |  | **** |  |
| 4 |  |  | **** | **** |
| 5 |  |  | **** | **** |
| 6 |  |  | **** | **** |
| 8 |  |  | **** | **** |
| 12 |  |  |  | **** |

G. Germination index rate (GRI)

| \| **Seeds storage time**  **(months)** \| \| --- \| | \| **1** \| \| --- \| | \| **2** \| \| --- \| | \| **3** \| \| --- \| | \| **4** \| \| --- \| |
| --- | --- | --- | --- | --- | --- | --- | --- | --- | --- |
| 1 | **** |  |  |  |
| 2 |  | **** |  |  |
| 3 |  | **** |  |  |
| 4 |  | **** | **** |  |
| 5 |  |  | **** |  |
| 6 |  | **** | **** |  |
| 8 |  |  | **** | **** |
| 12 |  |  |  | **** |
