# Supplementary material for: The Morphological Features and Biology of a Relict and Endangered Woody Plant Species: Chamaedaphne calyculata (L.) Moench (Ericaceae)
Source: Plants (Basel). 2019 May 15;8(5):129. doi: 10.3390/plants8050129 (PMC6572642; doi:10.3390/plants8050129)
Supplement: Supplementary file 1 [file plants-08-00129-s001.zip › Table S7.docx]

**Table S7**. Homogenous groups of means of the seed germination dynamics parameters determined on the basis of the Tukey HSD test (α = 0.05) with seeds storage temperature factor.

A. Germination percentage (GP)

| \| Seeds storage temperature  (°C) \| \| --- \| | \| 1 \| \| --- \| | \| 2 \| \| --- \| | \| 3 \| \| --- \| | \| 4 \| \| --- \| |
| --- | --- | --- | --- | --- | --- | --- | --- | --- | --- |
| 0-2 | **** |  |  |  |
| 2-4 |  | **** |  |  |
| 6-8 |  |  | **** |  |
| 21-23 |  |  |  | **** |

B. Time to first observed germinant (T)

| \| Seeds storage temperature  (°C) \| \| --- \| | \| 1 \| \| --- \| | \| 2 \| \| --- \| | \| 3 \| \| --- \| |
| --- | --- | --- | --- | --- | --- | --- | --- |
| 0-2 | **** |  |  |
| 2-4 | **** |  |  |
| 6-8 |  | **** |  |
| 21-23 |  |  | **** |

C. Time to maximum germination (T_100_)

| \| Seeds storage temperature  (°C) \| \| --- \| | \| 1 \| \| --- \| |
| --- | --- | --- | --- |
| 0-2 | **** |
| 2-4 | **** |
| 6-8 | **** |
| 21-23 | **** |

D. Mean germination time (MGT)

| \| Seeds storage temperature  (°C) \| \| --- \| | \| 1 \| \| --- \| | \| 2 \| \| --- \| |
| --- | --- | --- | --- | --- | --- |
| 0-2 | **** |  |
| 2-4 | **** |  |
| 6-8 | **** |  |
| 21-23 |  | **** |

E. Mean germination rate (MR)

| \| Seeds storage temperature  (°C) \| \| --- \| | \| 1 \| \| --- \| | \| 2 \| \| --- \| |
| --- | --- | --- | --- | --- | --- |
| 0-2 | **** |  |
| 2-4 | **** |  |
| 6-8 | **** |  |
| 21-23 |  | **** |

F. Germination index (GI)

| \| Seeds storage temperature  (°C) \| \| --- \| | \| 1 \| \| --- \| | \| 2 \| \| --- \| |
| --- | --- | --- | --- | --- | --- |
| 0-2 | **** |  |
| 2-4 | **** |  |
| 6-8 |  | **** |
| 21-23 |  | **** |

G. Germination index rate (GRI)

| \| Seeds storage temperature  (°C) \| \| --- \| | \| 1 \| \| --- \| | \| 2 \| \| --- \| | \| 3 \| \| --- \| | \| 4 \| \| --- \| |
| --- | --- | --- | --- | --- | --- | --- | --- | --- | --- |
| 0-2 | **** |  |  |  |
| 2-4 |  | **** |  |  |
| 6-8 |  |  | **** |  |
| 21-23 |  |  |  | **** |
