# Supplementary material for: The Morphological Features and Biology of a Relict and Endangered Woody Plant Species: Chamaedaphne calyculata (L.) Moench (Ericaceae)
Source: Plants (Basel). 2019 May 15;8(5):129. doi: 10.3390/plants8050129 (PMC6572642; doi:10.3390/plants8050129)
Supplement: Supplementary file 1 [file plants-08-00129-s001.zip › Table S8.docx]

**Table S8**. Homogenous groups of means of the seed germination dynamics parameters determined on the basis of the Tukey HSD test (α = 0.05) with two factors: seeds storage time and seeds storage temperature.

A. Germination percentage (GP)

| \| Seeds  storage time  (months) \| \| --- \| | \| Seeds storage  temperature  (˚C) \| \| --- \| | \| 1 \| \| --- \| | \| 2 \| \| --- \| | \| 3 \| \| --- \| | \| 4 \| \| --- \| | \| 5 \| \| --- \| | \| 6 \| \| --- \| | \| 7 \| \| --- \| | \| 8 \| \| --- \| | \| 9 \| \| --- \| | \| 10 \| \| --- \| | \| 11 \| \| --- \| | \| 12 \| \| --- \| | \| 13 \| \| --- \| | \| 14 \| \| --- \| |
| --- | --- | --- | --- | --- | --- | --- | --- | --- | --- | --- | --- | --- | --- | --- | --- | --- | --- | --- | --- | --- | --- | --- | --- | --- | --- | --- | --- | --- | --- | --- | --- |
| 1 | 0-2 |  |  |  |  |  |  |  |  |  |  |  |  |  | **** |
| 1 | 2-4 |  |  |  |  |  |  |  |  |  |  |  |  | **** | **** |
| 1 | 6-8 |  |  | **** | **** | **** | **** | **** | **** |  |  |  |  |  |  |
| 1 | 21-23 |  | **** | **** | **** | **** | **** | **** |  |  |  |  |  |  |  |
| 2 | 0-2 |  |  |  |  |  |  |  |  |  |  |  | **** | **** |  |
| 2 | 2-4 |  |  |  |  |  |  |  |  |  |  | **** | **** |  |  |
| 2 | 6-8 |  |  | **** | **** | **** | **** | **** |  |  |  |  |  |  |  |
| 2 | 21-23 | **** | **** | **** | **** | **** |  |  |  |  |  |  |  |  |  |
| 3 | 0-2 |  |  |  |  |  |  |  |  |  | **** | **** | **** |  |  |
| 3 | 2-4 |  |  |  |  |  |  |  | **** | **** | **** | **** |  |  |  |
| 3 | 6-8 |  | **** | **** | **** | **** | **** |  |  |  |  |  |  |  |  |
| 3 | 21-23 | **** | **** | **** | **** |  |  |  |  |  |  |  |  |  |  |
| 4 | 0-2 |  |  |  |  |  |  |  |  | **** | **** | **** |  |  |  |
| 4 | 2-4 |  |  |  |  | **** | **** | **** | **** | **** | **** |  |  |  |  |
| 4 | 6-8 | **** | **** | **** | **** | **** |  |  |  |  |  |  |  |  |  |
| 4 | 21-23 | **** | **** | **** | **** |  |  |  |  |  |  |  |  |  |  |
| 5 | 0-2 |  |  |  |  |  |  |  |  | **** | **** | **** |  |  |  |
| 5 | 2-4 |  |  |  | **** | **** | **** | **** | **** | **** |  |  |  |  |  |
| 5 | 6-8 |  | **** | **** | **** | **** | **** |  |  |  |  |  |  |  |  |
| 5 | 21-23 | **** | **** | **** |  |  |  |  |  |  |  |  |  |  |  |
| 6 | 0-2 |  |  |  |  |  |  | **** | **** | **** | **** | **** |  |  |  |
| 6 | 2-4 |  |  |  |  | **** | **** | **** | **** | **** | **** |  |  |  |  |
| 6 | 6-8 |  | **** | **** | **** | **** | **** |  |  |  |  |  |  |  |  |
| 6 | 21-23 | **** | **** | **** |  |  |  |  |  |  |  |  |  |  |  |
| 8 | 0-2 |  |  |  |  | **** | **** | **** | **** | **** | **** |  |  |  |  |
| 8 | 2-4 |  |  |  |  |  | **** | **** | **** | **** | **** |  |  |  |  |
| 8 | 6-8 | **** | **** | **** | **** | **** |  |  |  |  |  |  |  |  |  |
| 8 | 21-23 | **** | **** |  |  |  |  |  |  |  |  |  |  |  |  |
| 12 | 0-2 |  |  |  | **** | **** | **** | **** | **** | **** |  |  |  |  |  |
| 12 | 2-4 |  |  | **** | **** | **** | **** | **** | **** |  |  |  |  |  |  |
| 12 | 6-8 | **** | **** | **** | **** |  |  |  |  |  |  |  |  |  |  |
| 12 | 21-23 | **** |  |  |  |  |  |  |  |  |  |  |  |  |  |

B. Time to first observed germinant (T)

| \| Storage time  (months) \| \| --- \| | \| Storage  temperature  (˚C) \| \| --- \| | \| 1 \| \| --- \| | \| 2 \| \| --- \| | \| 3 \| \| --- \| | \| 4 \| \| --- \| | \| 5 \| \| --- \| | \| 6 \| \| --- \| |
| --- | --- | --- | --- | --- | --- | --- | --- | --- | --- | --- | --- | --- | --- | --- | --- |
| 1 | 0-2 | **** | **** | **** |  |  |  |
| 1 | 2-4 | **** | **** | **** | **** | **** |  |
| 1 | 6-8 | **** | **** | **** | **** | **** | **** |
| 1 | 21-23 |  |  |  | **** | **** | **** |
| 2 | 0-2 | **** | **** | **** |  |  |  |
| 2 | 2-4 | **** | **** | **** |  |  |  |
| 2 | 6-8 | **** | **** | **** | **** | **** | **** |
| 2 | 21-23 |  |  |  |  |  | **** |
| 3 | 0-2 | **** |  |  |  |  |  |
| 3 | 2-4 | **** |  |  |  |  |  |
| 3 | 6-8 | **** | **** | **** | **** | **** | **** |
| 3 | 21-23 |  | **** | **** | **** | **** | **** |
| 4 | 0-2 | **** | **** |  |  |  |  |
| 4 | 2-4 | **** | **** | **** |  |  |  |
| 4 | 6-8 | **** | **** | **** | **** | **** |  |
| 4 | 21-23 |  |  | **** | **** | **** | **** |
| 5 | 0-2 | **** | **** | **** |  |  |  |
| 5 | 2-4 | **** | **** | **** |  |  |  |
| 5 | 6-8 | **** | **** | **** | **** | **** |  |
| 5 | 21-23 |  |  |  |  | **** | **** |
| 6 | 0-2 | **** | **** | **** |  |  |  |
| 6 | 2-4 | **** | **** | **** |  |  |  |
| 6 | 6-8 | **** | **** | **** | **** |  |  |
| 6 | 21-23 |  |  | **** | **** | **** | **** |
| 8 | 0-2 | **** | **** | **** | **** | **** |  |
| 8 | 2-4 | **** | **** | **** | **** | **** |  |
| 8 | 6-8 | **** | **** | **** | **** | **** | **** |
| 8 | 21-23 |  |  | **** | **** | **** | **** |
| 12 | 0-2 | **** | **** | **** | **** |  |  |
| 12 | 2-4 | **** | **** | **** | **** | **** | **** |
| 12 | 6-8 | **** | **** | **** | **** | **** | **** |
| 12 | 21-23 |  |  |  | **** | **** | **** |

C. Time to maximum germination (T_100_)

| \| Storage time  (months) \| \| --- \| | \| Storage  temperature  (˚C) \| \| --- \| | \| 1 \| \| --- \| |
| --- | --- | --- | --- | --- | --- |
| 1 | 0-2 | **** |
| 1 | 2-4 | **** |
| 1 | 6-8 | **** |
| 1 | 21-23 | **** |
| 2 | 0-2 | **** |
| 2 | 2-4 | **** |
| 2 | 6-8 | **** |
| 2 | 21-23 | **** |
| 3 | 0-2 | **** |
| 3 | 2-4 | **** |
| 3 | 6-8 | **** |
| 3 | 21-23 | **** |
| 4 | 0-2 | **** |
| 4 | 2-4 | **** |
| 4 | 6-8 | **** |
| 4 | 21-23 | **** |
| 5 | 0-2 | **** |
| 5 | 2-4 | **** |
| 5 | 6-8 | **** |
| 5 | 21-23 | **** |
| 6 | 0-2 | **** |
| 6 | 2-4 | **** |
| 6 | 6-8 | **** |
| 6 | 21-23 | **** |
| 8 | 0-2 | **** |
| 8 | 2-4 | **** |
| 8 | 6-8 | **** |
| 8 | 21-23 | **** |
| 12 | 0-2 | **** |
| 12 | 2-4 | **** |
| 12 | 6-8 | **** |
| 12 | 21-23 | **** |

D. Mean germination time (MGT)

| \| Storage time  (months) \| \| --- \| | \| Storage  temperature  (˚C) \| \| --- \| | \| 1 \| \| --- \| | \| 2 \| \| --- \| | \| 3 \| \| --- \| | \| 4 \| \| --- \| |
| --- | --- | --- | --- | --- | --- | --- | --- | --- | --- | --- | --- |
| 1 | 0-2 | **** | **** | **** | **** |
| 1 | 2-4 | **** | **** | **** | **** |
| 1 | 6-8 | **** | **** | **** | **** |
| 1 | 21-23 |  |  | **** | **** |
| 2 | 0-2 | **** | **** | **** | **** |
| 2 | 2-4 | **** | **** | **** | **** |
| 2 | 6-8 | **** | **** | **** | **** |
| 2 | 21-23 |  | **** | **** | **** |
| 3 | 0-2 | **** | **** |  |  |
| 3 | 2-4 | **** | **** | **** | **** |
| 3 | 6-8 | **** | **** | **** | **** |
| 3 | 21-23 | **** | **** | **** | **** |
| 4 | 0-2 | **** | **** |  |  |
| 4 | 2-4 | **** | **** | **** |  |
| 4 | 6-8 | **** | **** | **** |  |
| 4 | 21-23 |  | **** | **** | **** |
| 5 | 0-2 | **** | **** | **** |  |
| 5 | 2-4 | **** | **** | **** |  |
| 5 | 6-8 | **** | **** | **** | **** |
| 5 | 21-23 |  |  |  | **** |
| 6 | 0-2 | **** |  |  |  |
| 6 | 2-4 | **** | **** | **** |  |
| 6 | 6-8 | **** | **** |  |  |
| 6 | 21-23 | **** | **** | **** | **** |
| 8 | 0-2 | **** |  |  |  |
| 8 | 2-4 | **** |  |  |  |
| 8 | 6-8 | **** | **** | **** |  |
| 8 | 21-23 | **** | **** | **** | **** |
| 12 | 0-2 | **** | **** |  |  |
| 12 | 2-4 | **** | **** | **** |  |
| 12 | 6-8 | **** | **** | **** | **** |
| 12 | 21-23 |  |  | **** | **** |

E. Mean germination rate (MR)

| \| Storage time  (months) \| \| --- \| | \| Storage  temperature  (˚C) \| \| --- \| | \| 1 \| \| --- \| | \| 2 \| \| --- \| | \| 3 \| \| --- \| | \| 4 \| \| --- \| | \| 5 \| \| --- \| |
| --- | --- | --- | --- | --- | --- | --- | --- | --- | --- | --- | --- | --- | --- |
| 1 | 0-2 | **** | **** | **** | **** | **** |
| 1 | 2-4 | **** | **** | **** | **** | **** |
| 1 | 6-8 | **** | **** | **** | **** | **** |
| 1 | 21-23 | **** | **** |  |  |  |
| 2 | 0-2 | **** | **** | **** | **** | **** |
| 2 | 2-4 | **** | **** | **** | **** | **** |
| 2 | 6-8 | **** | **** | **** | **** | **** |
| 2 | 21-23 | **** | **** | **** |  |  |
| 3 | 0-2 |  |  |  | **** | **** |
| 3 | 2-4 | **** | **** | **** | **** | **** |
| 3 | 6-8 | **** | **** | **** | **** | **** |
| 3 | 21-23 | **** | **** | **** | **** | **** |
| 4 | 0-2 |  |  |  | **** | **** |
| 4 | 2-4 |  | **** | **** | **** | **** |
| 4 | 6-8 | **** | **** | **** | **** | **** |
| 4 | 21-23 | **** | **** | **** | **** |  |
| 5 | 0-2 | **** | **** | **** | **** | **** |
| 5 | 2-4 | **** | **** | **** | **** | **** |
| 5 | 6-8 | **** | **** | **** | **** | **** |
| 5 | 21-23 | **** |  |  |  |  |
| 6 | 0-2 |  |  |  |  | **** |
| 6 | 2-4 | **** | **** | **** | **** | **** |
| 6 | 6-8 |  |  | **** | **** | **** |
| 6 | 21-23 | **** | **** | **** | **** | **** |
| 8 | 0-2 |  |  |  | **** | **** |
| 8 | 2-4 |  |  |  | **** | **** |
| 8 | 6-8 | **** | **** | **** | **** | **** |
| 8 | 21-23 | **** | **** | **** | **** | **** |
| 12 | 0-2 |  |  | **** | **** | **** |
| 12 | 2-4 | **** | **** | **** | **** | **** |
| 12 | 6-8 | **** | **** | **** | **** | **** |
| 12 | 21-23 | **** | **** |  |  |  |

F. Germination index (GI)

| \| Storage time  (months) \| \| --- \| | \| Storage  temperature  (˚C) \| \| --- \| | \| 1 \| \| --- \| | \| 2 \| \| --- \| | \| 3 \| \| --- \| | \| 4 \| \| --- \| | \| 5 \| \| --- \| | \| 6 \| \| --- \| |
| --- | --- | --- | --- | --- | --- | --- | --- | --- | --- | --- | --- | --- | --- | --- | --- |
| 1 | 0-2 |  |  |  |  |  | **** |
| 1 | 2-4 |  |  |  |  | **** | **** |
| 1 | 6-8 | **** | **** | **** |  |  |  |
| 1 | 21-23 |  | **** | **** |  |  |  |
| 2 | 0-2 |  |  |  |  | **** | **** |
| 2 | 2-4 |  |  |  | **** | **** |  |
| 2 | 6-8 | **** | **** | **** |  |  |  |
| 2 | 21-23 | **** | **** | **** |  |  |  |
| 3 | 0-2 |  |  | **** | **** |  |  |
| 3 | 2-4 |  |  | **** | **** |  |  |
| 3 | 6-8 | **** | **** | **** |  |  |  |
| 3 | 21-23 | **** | **** | **** |  |  |  |
| 4 | 0-2 |  |  | **** | **** |  |  |
| 4 | 2-4 | **** | **** | **** |  |  |  |
| 4 | 6-8 | **** | **** | **** |  |  |  |
| 4 | 21-23 | **** | **** | **** |  |  |  |
| 5 | 0-2 |  |  | **** | **** |  |  |
| 5 | 2-4 | **** | **** | **** |  |  |  |
| 5 | 6-8 | **** | **** | **** |  |  |  |
| 5 | 21-23 | **** | **** | **** |  |  |  |
| 6 | 0-2 |  | **** | **** |  |  |  |
| 6 | 2-4 |  | **** | **** |  |  |  |
| 6 | 6-8 | **** | **** | **** |  |  |  |
| 6 | 21-23 | **** | **** | **** |  |  |  |
| 8 | 0-2 | **** | **** | **** |  |  |  |
| 8 | 2-4 | **** | **** | **** |  |  |  |
| 8 | 6-8 | **** | **** | **** |  |  |  |
| 8 | 21-23 | **** | **** |  |  |  |  |
| 12 | 0-2 | **** | **** | **** |  |  |  |
| 12 | 2-4 | **** | **** | **** |  |  |  |
| 12 | 6-8 | **** | **** | **** |  |  |  |
| 12 | 21-23 | **** |  |  |  |  |  |

G. Germination index rate (GRI)

| \| Storage time  (months) \| \| --- \| | \| Storage  temperature  (˚C) \| \| --- \| | \| 1 \| \| --- \| | \| 2 \| \| --- \| | \| 3 \| \| --- \| | \| 4 \| \| --- \| | \| 5 \| \| --- \| | \| 6 \| \| --- \| | \| 7 \| \| --- \| | \| 8 \| \| --- \| | \| 9 \| \| --- \| | \| 10 \| \| --- \| | \| 11 \| \| --- \| | \| 12 \| \| --- \| | \| 13 \| \| --- \| | \| 14 \| \| --- \| |
| --- | --- | --- | --- | --- | --- | --- | --- | --- | --- | --- | --- | --- | --- | --- | --- | --- | --- | --- | --- | --- | --- | --- | --- | --- | --- | --- | --- | --- | --- | --- | --- |
| 1 | 0-2 |  |  |  |  |  |  |  |  |  |  |  |  |  | **** |
| 1 | 2-4 |  |  |  |  |  |  |  |  |  |  |  |  | **** | **** |
| 1 | 6-8 |  | **** | **** | **** | **** | **** |  |  |  |  |  |  |  |  |
| 1 | 21-23 | **** | **** | **** | **** |  |  |  |  |  |  |  |  |  |  |
| 2 | 0-2 |  |  |  |  |  |  |  |  |  |  |  | **** | **** |  |
| 2 | 2-4 |  |  |  |  |  |  |  |  |  | **** | **** | **** | **** |  |
| 2 | 6-8 |  | **** | **** | **** | **** | **** |  |  |  |  |  |  |  |  |
| 2 | 21-23 | **** | **** | **** |  |  |  |  |  |  |  |  |  |  |  |
| 3 | 0-2 |  |  |  |  |  |  |  |  |  |  |  |  | **** | **** |
| 3 | 2-4 |  |  |  |  |  |  |  | **** | **** | **** | **** | **** | **** |  |
| 3 | 6-8 |  | **** | **** | **** | **** | **** |  |  |  |  |  |  |  |  |
| 3 | 21-23 | **** | **** | **** |  |  |  |  |  |  |  |  |  |  |  |
| 4 | 0-2 |  |  |  |  |  |  |  |  |  |  | **** | **** | **** |  |
| 4 | 2-4 |  |  |  |  |  |  | **** | **** | **** | **** | **** | **** |  |  |
| 4 | 6-8 |  | **** | **** | **** | **** | **** | **** |  |  |  |  |  |  |  |
| 4 | 21-23 | **** | **** | **** |  |  |  |  |  |  |  |  |  |  |  |
| 5 | 0-2 |  |  |  |  |  |  |  |  | **** | **** | **** | **** | **** |  |
| 5 | 2-4 |  |  |  | **** | **** | **** | **** | **** | **** | **** |  |  |  |  |
| 5 | 6-8 |  | **** | **** | **** | **** | **** | **** |  |  |  |  |  |  |  |
| 5 | 21-23 | **** | **** |  |  |  |  |  |  |  |  |  |  |  |  |
| 6 | 0-2 |  |  |  |  |  |  |  |  |  | **** | **** | **** | **** |  |
| 6 | 2-4 |  |  |  |  |  | **** | **** | **** | **** | **** | **** |  |  |  |
| 6 | 6-8 |  |  | **** | **** | **** | **** | **** | **** | **** |  |  |  |  |  |
| 6 | 21-23 | **** | **** |  |  |  |  |  |  |  |  |  |  |  |  |
| 8 | 0-2 |  |  |  |  |  |  | **** | **** | **** | **** | **** | **** |  |  |
| 8 | 2-4 |  |  |  |  |  |  |  | **** | **** | **** | **** | **** |  |  |
| 8 | 6-8 |  | **** | **** | **** | **** |  |  |  |  |  |  |  |  |  |
| 8 | 21-23 | **** | **** |  |  |  |  |  |  |  |  |  |  |  |  |
| 12 | 0-2 |  |  |  |  | **** | **** | **** | **** | **** | **** | **** |  |  |  |
| 12 | 2-4 |  |  | **** | **** | **** | **** | **** | **** |  |  |  |  |  |  |
| 12 | 6-8 | **** | **** | **** |  |  |  |  |  |  |  |  |  |  |  |
| 12 | 21-23 | **** |  |  |  |  |  |  |  |  |  |  |  |  |  |
