# Supplementary material for: The Morphological Features and Biology of a Relict and Endangered Woody Plant Species: Chamaedaphne calyculata (L.) Moench (Ericaceae)
Source: Plants (Basel). 2019 May 15;8(5):129. doi: 10.3390/plants8050129 (PMC6572642; doi:10.3390/plants8050129)
Supplement: Supplementary file 1 [file plants-08-00129-s001.zip › Table S9.docx]

**Table S9**. Variability of morphological features of assimilating leaves leaf of the examined population of *Ch. calyculata* (“New population”) versus literature data [5, 26, 27] based on the coefficient of variation of the mean (CV)

|  | Length of leaf blade | Leaf blade width | Length of petiole | Leaf blade length-to-width ratio | Half of the angle at the leaf blade base | Half of the angle at the leaf blade apex | The average  coefficient  of variation  of the mean (CV)  per population |
| --- | --- | --- | --- | --- | --- | --- | --- |
| Krasnopol | 15.09 | 13.76 | 15.79 | 10.06 | 19.55 | 15.51 | **14.96** |
| Jesionowe Góry | 10.84 | 10.85 | 21.74 | 10.14 | 17.87 | 12.24 | **13.95** |
| Gorbacz | 16.67 | 18.06 | 23.53 | 10.43 | 21.81 | 13.43 | **17.32** |
| Lisie Jamy | 12.93 | 15.38 | 22.22 | 15.69 | 17.85 | 17.39 | **16.91** |
| Krutyn | 13.45 | 12.94 | 25.00 | 13.53 | 17.65 | 17.16 | **16.62** |
| Sołtysek | 14.61 | 14.61 | 30.00 | 12.07 | 21.23 | 19.15 | **18.61** |
| Sieraków | 10.17 | 13.33 | 21.05 | 8.40 | 13.71 | 10.26 | **12.82** |
| Sowiniec - small leaf blade | 13.85 | 14.08 | 25.00 | 16.06 | 15.76 | 18.77 | **17.25** |
| Sowiniec - large leaf blade | 24.65 | 22.22 | 28.57 | 11.79 | 15.76 | 14.39 | **19.56** |
| Sitno | 7.89 | 11.22 | 22.22 | 9.03 | 19.35 | 14.61 | **14.05** |
| Babagniewa | 13.72 | 15.48 | 28.57 | 11.76 | 14.27 | 12.60 | **16.07** |
| Archangielsk | 13.76 | 15.63 | 23.08 | 15.11 | 14.69 | 16.67 | **16.49** |
| **General 1-12** | 14.04 | 13.00 | 23.53 | 11.64 | 13.14 | 12.00 | **14.56** |
| **New population** | **16.74** | **14.10** | **35.05** | **10.55** | **19.14** | **19.63** | **19.20** |
